# Impact of early chimerism status on clinical outcome in children with acute lymphoblastic leukaemia after haematopoietic stem cell transplantation

**DOI:** 10.1186/s12885-019-6360-3

**Published:** 2019-11-26

**Authors:** Monika Lejman, Agnieszka Zaucha-Prażmo, Joanna Zawitkowska, Aleksandra Mroczkowska, Dominik Grabowski, Jerzy R. Kowalczyk, Katarzyna Drabko

**Affiliations:** 1 Laboratory of Genetic Diagnostics, Department of Pediatric Hematology, Oncology, and Transplantology, Medical University of Lublin, A. Gebali 6, 20-093 Lublin, Poland; 2 Department of Pediatric Hematology, Oncology, and Transplantology, Medical University of Lublin, A. Gebali 6, 20-093 Lublin, Poland

**Keywords:** Chimerism, Engraftment, Quantitative PCR, GvHD, Acute lymphoblastic leukaemia, Allogeneic Haematopoietic stem cell transplantation

## Abstract

**Background:**

The significance of very early chimerism assessment before day + 28, which is considered the moment of engraftment, is still unclear. In this retrospective study, we evaluated the clinical impact of very early chimerism on the clinical outcome after allogeneic haematopoietic stem cell transplantation (allo-HSCT) in children with acute lymphoblastic leukaemia (ALL).

**Methods:**

The study group included 38 boys and 18 girls. Very early chimerism was evaluated on days + 7, + 14, + 21 and + 28 after the transplant. Short tandem repeat polymerase chain reaction (STR PCR) was used to analyse chimerism.

**Results:**

Overall survival (OS) and event-free survival (EFS) were 84 and 80%, respectively. The OS in the group of 24 patients with complete donor chimerism on day + 14 was 83%, and it did not differ statistically compared to the 32 patients with mixed chimerism on day + 14 (OS was 84%). In our cohort of patients, the matched unrelated donor, male gender of donor, number of transplanted cells above 4.47 × 10^6^ kg and no serotherapy with anti-thymocyte globulin (ATG) were statistically related to a higher level of donor chimerism. The immunophenotypes of disease, age of patient at time HSCT, recipient sex, stem cell source (peripheral blood/bone marrow) and conditioning regimen had no impact on early chimerism. Acute graft versus host disease grades II-IV was diagnosed in 23 patients who presented with donor chimerism levels above 60% on day 7.

**Conclusions:**

The data presented in this study provide valuable insight into the analysis of very early chimerism in children with ALL treated with HSCT.

## Background

Current chemotherapy regimens for acute lymphoblastic leukaemia (ALL) result in a remission in the majority of children with the disease. Despite remarkable improvement in the treatment of this malignancy, 20% of children still relapse, and their outcome remains poor [1]. Allogeneic haematopoietic stem cell transplantation (allo-HSCT) for these children has become a well-established treatment to control the disease [2]. The curative effect of allogeneic HSCT for acute leukaemia is attributed to the graft versus leukaemia effect produced by allogeneic immune cells, as well as intensive conditioning chemotherapy with or without radiotherapy [3].

It is well known that chimerism monitoring is an important diagnostic tool for assessing the risk of relapse after allo-HSCT in patients with malignant diseases, especially in those, who lack specific markers for tracking residual disease [4]. However, the significance of very early chimerism assessment before day + 28, which is considered the moment of the engraftment, is still unclear. The studies show that early analysis of T- and NK-cell chimerism can therefore be instrumental in risk assessment and therapeutic management of imminent graft rejection [5].

Investigations on the new methods for routine chimerism monitoring are very promising; however, the current gold standard method of monitoring chimerism is short tandem repeat polymerase chain reaction (STR PCR), which not only determines the type of chimeras, but also determines the percentage of both donor and recipient cells [6–8]. The persistence or reappearance of recipient cells after allo-HSCT can indicate the presence of malignant cells or the recurrence of the recipient’s haematopoietic cells or a combination of both [9].

The aim of this study was to analyse the dynamics of early chimerism after allogeneic HSCT in children with ALL and its role in the assessment of survival and event-free survival. Furthermore, this study analyses the evolution of chimerism over time and evaluates the impact of transplant variables on chimerism.

## Methods

### Patients

The research encompassed biological material (peripheral blood) derived from 56 consecutive children diagnosed with acute lymphoblastic leukaemia who had undergone allogeneic haematopoietic stem cell transplantation at the Department of Paediatric Haematology, Oncology and Transplantology of Medical University in Lublin between 2002 and 2018. The patients’ characteristics are summarized in Table 1.

**Table 1:** Characteristics of patients and transplantation

	Patients *n* = 56 (100%)
Immunophenotype	
B ALL	31 (55%)
T ALL	25 (45%)
1 CR	26 (46%)
> 2 CR	30 (54%)
Median age at transplant (range) years	9,04 (1,72–17)
Patient gender	
Male	38 (68%)
Female	18 (32%)
Donor type	
Matched related	25 (45%)
Matched unrelated	28 (50%)
Mismatched related	3 (5%)
Donor gender	
Male	31 (55%)
Female	25 (45%)
Stem cell source	
Bone marrow	47 (84%)
Peripheral blood	9 (16%)
Conditioning regimen	
Radiation-base	41 (73%)
Busulfan-based	2 (3%)
Reduced toxicity	13 (24%)
Serotherapy (ATG)	
YES	28 (50%)
NO	28 (50%)
Number of CD34+ cells (median 4,47) range (2–13,3 × 10^6^/kg)	
< 4,47 × 10^6^	35 (62%)
> 4,47 × 10^6^	21 (38%)
aGvHD	23 (41%)
cGvHD	4 (7%)

*B ALL* B-cell acute lymphoblastic leukaemia, *T ALL* T-cell acute lymphoblastic leukaemia, *CR* complete remission, *ATG* Antithymocyte globulin, *aGvHD* Acute Graft Versus Host Disease, *cGvHD* Chronic Graft Versus Host Disease

All patients were conditioned according to the European Bone Marrow Transplantation (EBMT) guidelines [10]. Conditioning was myeloablative (MAC), and standard regimens were based on fractionated total body irradiation (FTBI) or busulfan. In reduced toxicity conditioning (RTC), treosulfan was used instead of busulfan. Cyclosporine was used as a graft versus host disease (GvHD) prophylaxis. Matched unrelated transplant recipients received anti-thymocyte globulin (ATG) to prevent GvHD. Mismatched related transplant recipients received ex-vivo T-cell depleted grafts. Engraftment was diagnosed when an absolute neutrophil count (ANC) of 500 or more was observed for 2 days.

### Chimerism analysis

Very early chimerism was evaluated from peripheral blood (PB) on days + 7, + 14, + 21 and + 28. Next, samples were collected, and chimerism was monitored according to the EBMT guidelines as a part of the routine follow-up post allo-HSCT [9, 10]. Depending on clinical indications, chimerism was monitored irrespective of the scheduled time points. A previously described STR PCR method that has been standardized in our laboratory based on Eurochimerism recommendations was used for chimerism assessment [11, 12]. The sensitivity of our method for detecting recipient cells was 1%, but patients with verified 1% autologous cells in 2 repeated samples were considered mixed chimeras. Early mixed chimerism was determined as the presence of 1% or more recipient cells in peripheral blood.

The genomic DNA was isolated from mononuclear peripheral blood cells. Peripheral blood was aspirated into anticoagulant (EDTA)-containing tubes. The isolation of the mononuclear cell (MNC) fraction was performed using Ficoll-Paque PLUS aqueous solution of 1.077 + 0.001 g/ml density (Amersham Biosciences, Inc., Piscataway, NJ, USA). DNA was isolated with the QIAamp DNA Blood Mini Kit (Qiagen, Hilden, Germany). Finally, DNA was eluted from the column with 60–70 μl of elution buffer. The details of the chimerism analysis method were described in our previous publication [11].

Statistical analysis was performed using SPSS IBM Statistics (Version 24) and XLSTAT 2019 1.3. Non-parametric tests (Pearson’s Chi-square, chi-square test with simulating *p* values – test insensitive to small numbers, Kruskal-Wallis) were used for group comparison. OS and EFS were estimated using Kaplan-Meier method and Log-rank tests. Cumulative incidence of relapse was performed using STATA. Statistical significance was considered < 0.05.

The study was approved by the Ethics Committee of the Medical University of Lublin (KE-0254/70/2010).

## Results

### The median follow-up was 4.58 years (1.00–15.79 years)

**Fig. 1:**
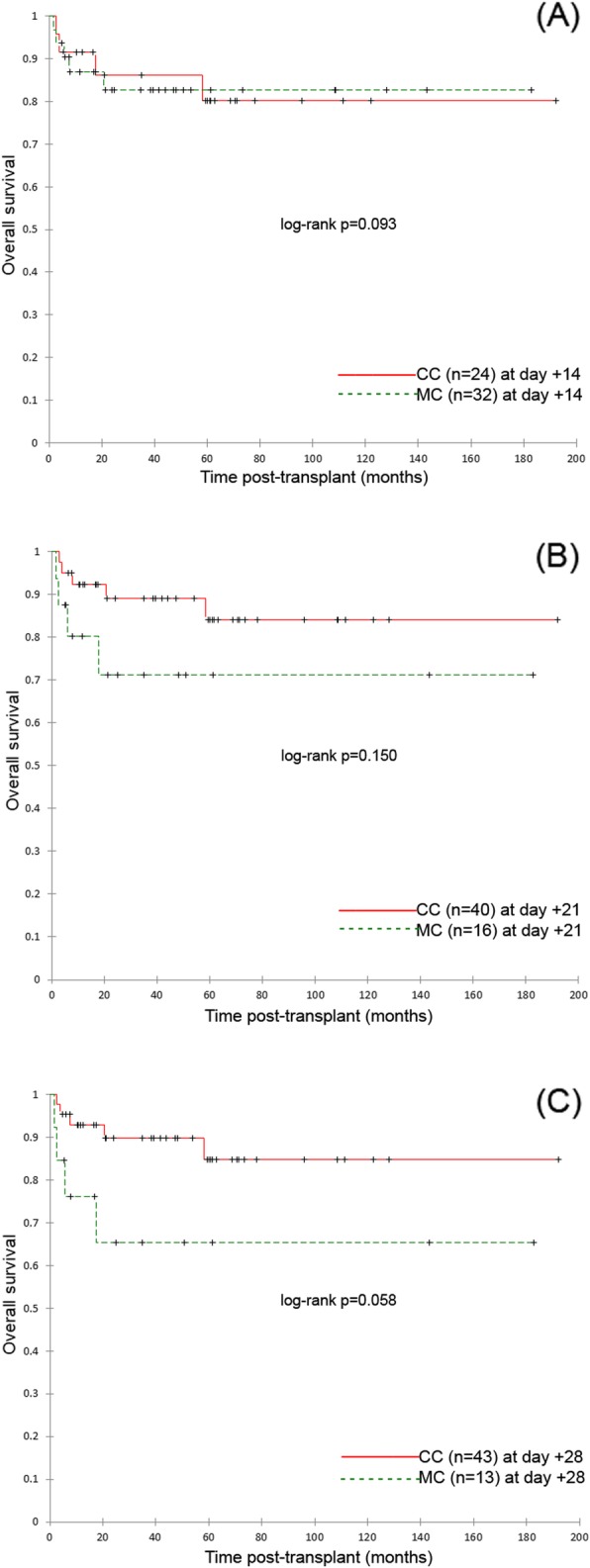
Overall survive (OS) of study patients. **a** Kaplan-Meier survival plots for OS of study patients with complete donor chimerism (*n* = 24) in day 14 and patients with mixed chimerism (*n* = 32) in day 14. Study cohort: *n* = 56, *p* = 0.093. **b** Kaplan-Meier survival plots for OS of study patients with complete donor chimerism (*n* = 40) in day 21 and patients with mixed chimerism (*n* = 16) in day 21. Study cohort: *n* = 56, *p* = 0.150. **c** Kaplan-Meier survival plots for OS of study patients with complete donor chimerism (*n* = 43) in day 28 and patients with mixed chimerism (*n* = 13) in day 28. Study cohort: *n* = 56, *p* = 0.058

**Fig. 2:**
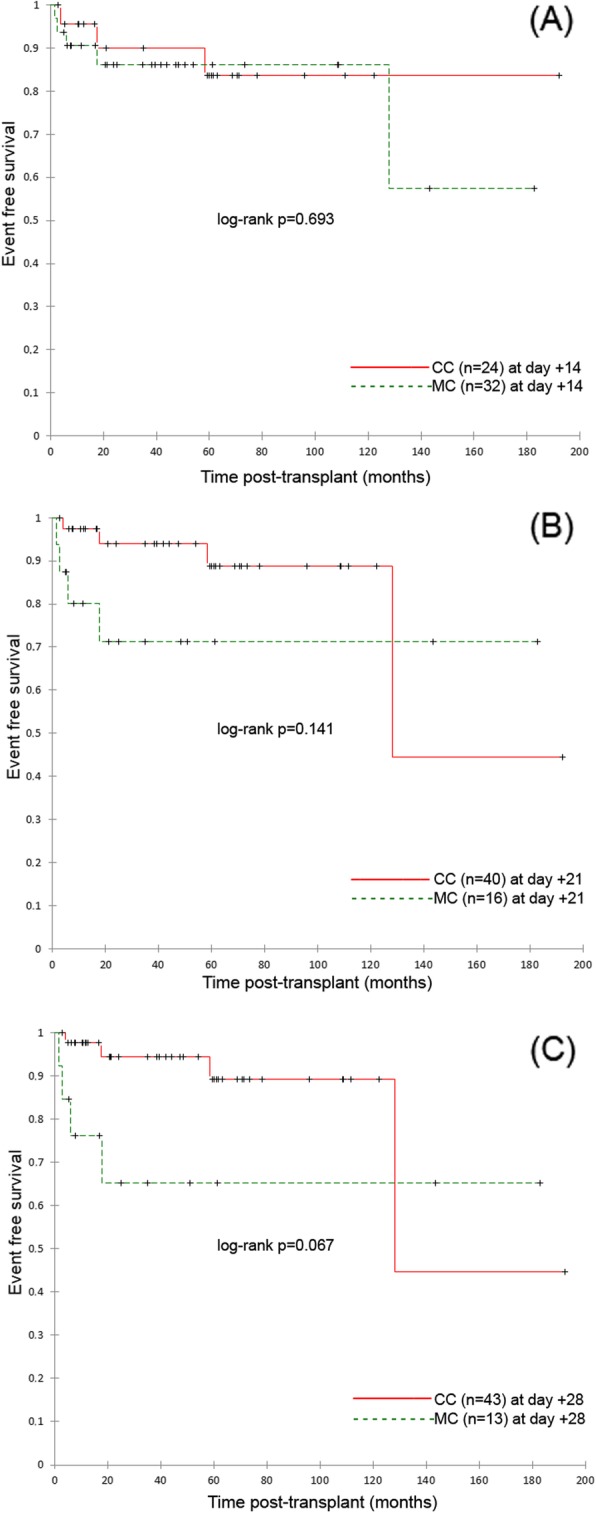
The even free survive (EFS) of study patients. **a** Kaplan-Meier survival plots for EFS of study patients with complete donor chimerism (*n* = 24) in day 14 and patients with mixed chimerism (*n* = 32) in day 14. Study cohort: *n* = 56, *p* = 0.693. **b** Kaplan-Meier survival plots for EFS of study patients with complete donor chimerism (*n* = 40) in day 21 and patients with mixed chimerism (*n* = 16) in day 21. Study cohort: *n* = 56, *p* = 0.141. **c** Kaplan-Meier survival plots for EFS of study patients with complete donor chimerism (*n* = 43) in day 28 and patients with mixed chimerism (*n* = 13) in day 28. Study cohort: *n* = 56, *p* = 0.067

The 5-year overall survival (OS) and event-free survival (EFS) for the whole group of patients were 84 and 80%, respectively. Further comparisons were performed in groups of children with complete chimerism (CC) and mixed chimerism (MC) assessed on days + 14, + 21, + 28. On day + 7 all, but one patient presented MC, therefore statistical analyses were not performed for this time point. No statistical differences were found in OS and EFS in analysed time points. The results are presented on Figs. 1 and 2.

**Fig. 3:**
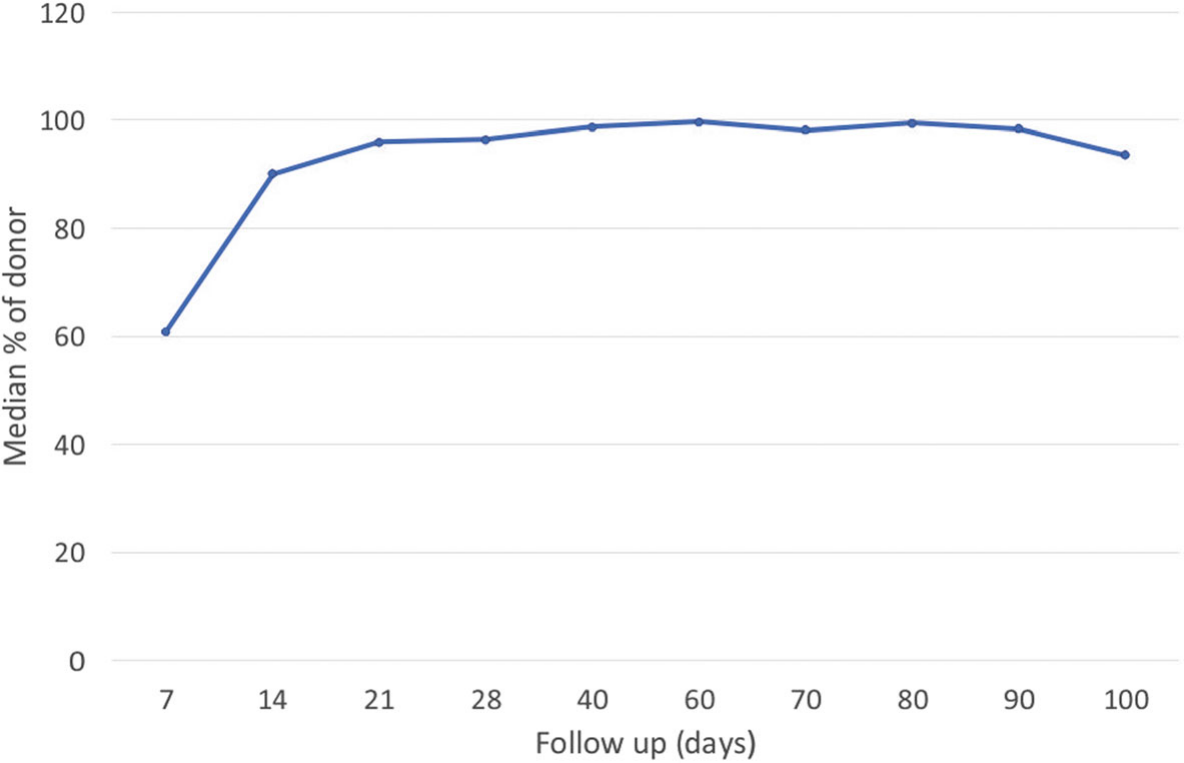
The graph shows the kinetics of chimerism in all analysed patients The results presented on the curve are median (expressed as a percentage) values of donor cells in each time point on the schedule of monitoring chimerism

Analysis of early chimerism showed that the median donor chimerism level was 60% on day + 7, 90% on day + 14, 96% on day + 21, and 98% on day + 28. The kinetics of early chimerism in the studied group of patients is presented in Fig. 3.

We analysed factors, that may have influenced the status of early chimerism (Table 2). In our cohort of patients, the matched unrelated donor, male donor, number of transplanted CD34+ cells above 4.47 × 10^6^ kg and no ATG serotherapy were statistically related to a higher level of donor chimerism. The immunophenotype of the disease, patient’s age at HSCT, recipient’s sex, stem cell source (peripheral blood/bone marrow) and conditioning regimen had no impact on early chimerism.

**Table 2:** Characteristics of factors influencing on the early chimerism status

Transplant variables	Donor chimerism level (median %)							
	+ 7 day	*p* value	+ 14 day	*p* value	+ 21 day	*p* value	+ 28 day	*p* value
Underlying diseases								
B ALL *n* = 31	58	*p* = 0.9	100	*p* = 0.3	100	*p* = 0.79	100	*p* = 0.58
T ALL *n* = 25	63		95		100		100	
Age of patient								
< median (8.67 years) *n* = 28	58	*p* = 0.98	95	*p* = 0.66	100	*p* = 0.73	100	*p* = 0.86
> median (8.67 years) *n* = 28	65		99		100		100	
Patient gender								
Male *n* = 38	61	*p* = 0.99	94	*p* = 0.15	100	*p* = 0.68	100	*p* = 0.7
Female *n* = 18	62		100		100		100	
Donor type								
Matched related *n* = 25	49	*p* = 0.06	90	***p*** **= 0.02**	100		100	
Matched unrelated *n* = 28	68		100		100	***p*** **= 0.01**	100	***p*** **= 0.02**
Mismatched related *n* = 3	80		100		100		100	
Donor gender								
Male *n* = 31	77	***p*** **= 0.003**	100	***p*** **= 0.041**	100	*p* = 0.56	100	*p* = 0.80
Female *n* = 25	46		89		100		100	
Stem cell source								
Bone marrow *n* = 47	60	*p* = 0.64	96	*p* = 0.20	100	*p* = 0.32	100	*p* = 0.19
Peripheral blood *n* = 9	63		100		100		100	
Conditioning regimen								
Radiation-based *n* = 41	60	*p* = 0.33	96	*p* = 0.40	100	*p* = 0.40	100	*p* = 0.43
Busulfan-based *n* = 2	74		80		85		91	
Reduced toxicity *n* = 13	67		100		100		100	
Serotherapy (ATG)								
YES *n* = 28	49	***p*** **= 0.05**	94	*p* = 0.11	100	***p*** **= 0.016**	100	***p*** **= 0.023**
NO *n* = 28	68		100		100		100	
Number of CD34+ cells (median 4,47)								
< 4,47 × 10^6^ *n* = 35	58	*p* = 0.41	94	***p*** **= 0.036**	100	*p* = 0.08	100	*p* = 0.19
> 4,47 × 10^6^ *n* = 21	67		100		100		100	
Patients								
without event *n* = 48	62	*p* = 0.98	68	*p* = 0.48	100	*p* = 0.88	100	*p* = 0.56
with relapse *n* = 5	64		97		100		100	
with TRM no relapse *n* = 3	65		81		100		100	

*B ALL* B-cell acute lymphoblastic leukaemia, *T ALL* T-cell acute lymphoblastic leukaemia, *ATG* Anti-thymocyte globulin, *TRM* Transplant related mortality, *p* = 0.05 this result is on the border of the statistical significance

Acute graft versus host disease (aGvHD) grades II-IV was diagnosed in 23 patients. For statistical analyses of the effect of donor chimerism levels on aGvHD incidence, all patients were divided into two groups based on donor chimerism levels above and below 80%. These values were determined on the basis of the fact that in the whole group of patients, the median percentage of donor chimerism before + 28 days reached 80%. No statistically significant effect of the level of donor chimerism above 80% achieved by patients before day + 28 on the incidence of aGvHD was found (*p* = 0.22 on day 7; *p* = 0.69 on day 14; *p* = 0.93 on day 21; *p* = 0.75 on day 28). It was found that in all patients who developed aGvHD, the level of donor chimerism on day + 7 was above 60%.

The level of donor chimerism above 80% had no effect on chronic graft versus host disease (cGvHD) (*p* = 0.05 on day 7; *p* = 0.93 on day 14; *p* = 0.85 on day 21; *p* = 0.27 on day 28).

In two patients, increasing recipient chimerism was found on day + 21, and for that reason, the cyclosporine was discontinued. These children have achieved complete donor chimerism on days + 40 and + 90, respectively. At the end of the observation period, both were alive and in complete remission 2.5 and 3.5 years, respectively, after HSCT with complete donor chimerism.

Forty-eight patients (86%) were alive and in complete remission (in 45 patients, complete donor chimerism was found, while recipient haematopoiesis was detected in three patients). Eight patients (14%) died. Relapse occurred in five of them (9%) after day + 28, between 3 months and 4.5 years post-HSCT. All relapsed patients presented with increasing recipient chimerism (IMC) on days + 91, + 93, + 331, + 444, and + 1285, respectively. The relapse was diagnosed between 7 and 10 days after IMC was diagnosed. Three of the relapsed patients achieved complete donor chimerism early on day + 14. No difference in cumulative incidence (CI) of relapse was observed in patients with donor chimerism lower and higher than 60% on day + 7: CI (95%) 0.114 (0.031–0.43) and 0.139 (0.05–0.406) respectively (*p* = 0.56); as well as in CI of relapse in patients with MC and CC on day + 14, respectively: CI (95%) 0.063 (0.015–0.28) and 0.161 (0.061–0.461), respectively (*p* = 0.35). Three patients (5%) died due to transplant-related complications.

## Discussion

The literature data suggest, that chimerism analyses are routinely performed for the surveillance of engraftment. In recent years, these studies have become the basis for therapeutic intervention [13, 14]. Analysis of short tandem repeats used in our study, is described in the literature with the recommendations for current laboratory practice as the method for the post-transplant monitoring of donor engraftment [12, 15]. The previous literature data suggested that serial mixed chimerism analysis in patients with acute laeukemia at the short time intervals by PCR provides a reliable and rapid screening method for the early detection of relapse [16]. Based on its limited sensitivity to detect a minor cell population of approximately 1%, monitoring of chimerism in the whole blood is not suitable to serve as the minimal residual disease (MRD) marker. For the assessment of MRD, other techniques should be used [17].

Relapse after transplantation is a major cause of treatment failure in paediatric patients with ALL. Barrios M et al. [18] presented results that indicate that constants assessments of chimerism allows the prediction of relapse and death after HSCT for acute leukaemia. In their study, patients with increasing mixed chimerism (IMC) showed a significantly higher (*p* < 0.001) rate of relapse (93.1%) and death (89.7%) in comparison to both those with complete chimerism (CC) (29.9% relapse, 44.1% dead) or decreasing MC (11.1% relapse, 44.4% dead). Relapse was found in 39.8% of analysed patients [18]. The correlation between donor chimerism status and disease relapse after allo-HSCT was investigated by Jiang Y et al. [19]. They showed, that a total of 21.6% of patients had recurrent disease. In our analysed group, relapse was observed in 9% of patients who showed increasing recipient chimerism, although on day + 14, they were complete chimeras. Five-year OS and 5-year EFS were 62.07 ± 4.37% and 56.17 ± 4.38%, respectively, for the overall cohort of patients with ALL in the Pachon C et al. study [20]. In our study, OS (83%) and EFS (84%) were higher than those described in the literature [20]. Three of our patients are alive with stable mixed chimerism (10% donor, 35% donor and 90% donor, respectively), which is in line with the observation of Levrat E. et al. with very long-term stability of MC in patients with haematologic malignancies [21].

Lassaletta A et al. [22] analysed chimerism by the day 30 after peripheral blood progenitor cell transplantation. A total of 27/39 patients showed CC by day 30 after HSCT, but the median time to achieve CC was 15 days (range 8–750). In 15.4% of patients, CC was never achieved [22]. On the day + 7, a median of 61% of our patients presented very early complete donor chimerism, whereas on the day + 14, a median of 90% patients had complete donor chimerism. Finally, 48/56 (86%) of our patients achieved complete donor chimerism.

Comparable to other studies, in our cohort no correlation between early donor chimerism and the source of stem cell transplantation was found, but the number of transplanted CD34+ cells had a significant impact on patients’ chimerism status [22, 23]. We observed that matched unrelated and male donors were connected with high-level donor chimerism on days + 7 and +  14, similar to other reports [24].

According to Sakellarii I at al. study, the survival rates in matched transplants were promising at a relatively low-dose ATG as an effective prophylaxis for acute GvHD [25]. Our results indicate that ATG is also effective but connected to mixed chimerism in the very early period after HSCT. In patients who received ATG as GvHD prophylaxis, engraftment with complete donor chimerism was observed later than that in patients without ATG (day + 21 vs + 14, respectively). The conditioning regimen (myeloablative) did not affect early chimerism status, which is compatible with other reports [26, 27].

Lassaletta et al. [22] presented results correlation between the chimerism status at + 30 day and chronic GvHD. They observed, that the status of CC by day + 30 after HSCT, was notably related to the development of chronic GvHD. Patients who presented CC by day + 30 had statistically higher, probability of developing chronic GvHD, comparing to the patients with MC by day + 30 [22]. Mossalam G et al. [26] observed that low donor chimerism in patients was connected with a reduced risk of the development chronic GvHD. Jaksch M et al. [28] noticed a significantly higher risk of aGvHD grades II-IV in patients with complete donor CD4+ T-cell chimerism on day 7 and patients who increased 50% or more in donor CD4+ T cells between days 7 and 10 after SCT. We did not observe a correlation between early donor chimerism and aGvHD or cGvHD, perhaps due to GvHD prophylaxis. The patients who developed cGvHD, presented donor chimerism above 80% on day + 7 (*p* = 0.05). To confirm this finding, the studies should be continued on a larger group of patients.

Horn B et al. [29] describe the long-term follow-up of children with acute laeukemia with early mixed chimerism-based post-transplant immunotherapy. Children receiving post-transplant immunotherapy had similar outcomes to patients achieving complete donor chimerism spontaneously. Rettinger E et al. added that the immunotherapy in the patients with mixed chimerism improves survival in childhood ALL and does not increase the risk of acute GvHD [30]. In our cohort, a successful therapeutic intervention was undertaken twice on the basis of chimerism measurements in the early post-transplant period (day + 21).

## Conclusions

The data presented in this study provide a valuable input for the analysis of the significance of the very early assessment of chimerism in children with ALL treated with HSCT. Our findings suggest that early monitoring of chimerism after HSCT may be a helpful tool in predicting transplant rejection and using successful therapeutic intervention. This study has its limitations, such as no evaluation of the chimerism in lymphocyte subpopulations. Prospective observational studies and multicentre retrospective studies on larger groups of patients, including those diagnosed with different malignancies, would allow comparison of the results obtained for different groups of patients.

## Data Availability

Data and material are available upon request. Agnieszka Zaucha-Prażmo e-mail agnieszkazauchaprazmo@umlub.pl

## Abbreviations

aGvHD: Acute Graft versus Host Disease
ALL: Acute Lymphoblastic Leukaemia
Allo-HSCT: Allogeneic Haematopoietic Stem Cell Transplantation
ANC: Absolute neutrophil count
ATG: Anti-thymocyte Globulin
CC: Complete chimerism
cGvHD: Chronic Graft versus Host Disease
CI: Cumulative incidence
EBMT: European Bone Marrow Transplantation
EFS: Event-Free Survival
GvHD: Graft versus Host Disease
IMC: Increasing mixed chimerism
MC: Mixed chimerism
MNC: Mononuclear cells
MRD: Minimal residual disease
OS: Overall Survival
PB: Peripheral blood
RTC: Reduced Toxicity Conditioning
STR PCR: Short Tandem Repeats Polymerase Chain Reaction
